# Characterisation of the complete mitochondrial genome of the Jinchuan Yak (*Bos grunniens*)

**DOI:** 10.1080/23802359.2019.1681312

**Published:** 2019-11-08

**Authors:** Pengjia Bao, Jie Pei, Xuezhi Ding, Xiaoyun Wu, Min Chu, Lin Xiong, Chunnian Liang, Xian Guo, Ping Yan

**Affiliations:** a Lanzhou Institute of Husbandry and Pharmaceutical Sciences, Chinese Academy of Agricultural Sciences, Lanzhou, China;; b Key Laboratory of Yak Breeding Engineering of Gansu Province, Lanzhou, China

**Keywords:** Jinchuan yak, mitochondrial genome, characterization

## Abstract

Jinchuan yak was a newly discovered yak breed, not only possesses a large proportion of multi-ribs but also exhibits many good characteristics. However, there is limited information about its overall genetic structure. In this study, we assembled the mitochondrial genome for Jinchuan yak (*Bos grunniens*), the results show that the mitochondrial genome is 16,324bp long with an A + T-biased base composition (61.0% A + T) and harbours the typical set of 37 mitochondrial genes and 1 non-coding control region. The PCGs start with the typical ATA or ATG codons and are terminated with TAA, TAG or the incomplete stop codon T. Phylogenetic analysis suggests that Jinchuan yak is most closely related to Datong yak and Sunan yak.

Jinchuan yak was an unique native yak breed, famous for its special anatomical characteristic: an additional pair of ribs compared to other yak breeds which only have 14 pairs of ribs (Mipam et al. 2012). It was found in recent years with special biological characteristics and better production performance. The genetic structure of this population is unknown. Here, the complete mitochondrial genome of Jinchuan yak was characterized by high-throughput Illumina sequencing technology. Besides, we also investigated its relationship with its congeners and the closely related genus Bison (Guo et al. 2019).

The blood sample of Jinchuan yak was collected from Jinchuan County, Maori country Reta village, Sichuan province (31°43′N, 101°52′E). A voucher specimen is held in the Key Laboratory of Yak Breeding Engineering of Gansu Province, Lanzhou Institute of Husbandry and Pharmaceutical Sciences (Lanzhou, Gansu Province, China). The genome DNA of Jinchuan yak was stored in the Genetic Resource Collection Room (GRCR) of our department at −80 °C, the store ID was No. 20190529. Isolation of genomic DNA was carried out with the QIAamp DNA Blood Mini Kit (Qiagen, CA, USA).

Library preparation and high-throughput sequencing were conducted by Xi’an Genedigger Biotechnology Co., Ltd. (Xi’an, China) following the manufacturer’s protocol of the Illumina HiSeq X Ten Sequencing System (Illumina, CA, USA). A total of 2.23 Gb raw data was used to assemble the mitochondrial genome with MITObim v1.9 (Hahn et al. 2013); the reference sequence was previously published by Qiu et al. (2012) (GenBank accession: JQ692071).

The mitochondrial genome of Jinchuan yak (GenBank accession: MN176980) is highly similar to those of other yak breeds (Qiu et al. 2012*;* Chu et al. 2016*;* Guo et al. 2016*;* Wu et al. 2016). The final size of the complete mitogenome of the Jinchuan yak was 16,324bp, the base composition was 33.7% A, 27.3% T, 13.2% G and 25.8% C, and harbours the typical set of 37 animal mitochondrial genes and 1 control region (D-Loop). The typical start codon ATA was annotated for three protein-coding genes (PCGs) (*ND2, ND3*, and *ND5*), and ATG for the remaining ten PCGs (*ATP6, ATP8, COX1, COX2, COX3, CYTB, ND1, ND4, ND4L,* and *ND6*) codons. Three types of stop codons were annotated, i.e. TAA (*ATP6, ATP8, COX1, COX2, CYTB, ND1, ND4L, ND5,* and *ND6*), TAG (*ND2*), and the incomplete stop codon T (*COX3, ND3,* and *ND4*). The tRNAs vary in size between 60 (*tRNA-Ser^AGY^*) and 75 bp (*tRNA-Leu^UUR^*) with a total length of 1513 bp. The *12S rRNA* is 957 bp and *16S rRNA* is 1571 bp long, they are separated by tRNA-Val. The control region (D-loop) is 894 bp long and resides between tRNA-Pro and tRNA-Phe. Phylogenetic analysis was conducted based on the maximum-likelihood analysis of the concatenated sequences of all 13 mitochondrial PCGs for a group of 30 Bos and Bison taxa with MEGA7 (Kumar et al. 2016) (Figure 1), the result shows that Jinchuan yak was most closely related to Datong yak and Sunan yak.

**Figure 1.**
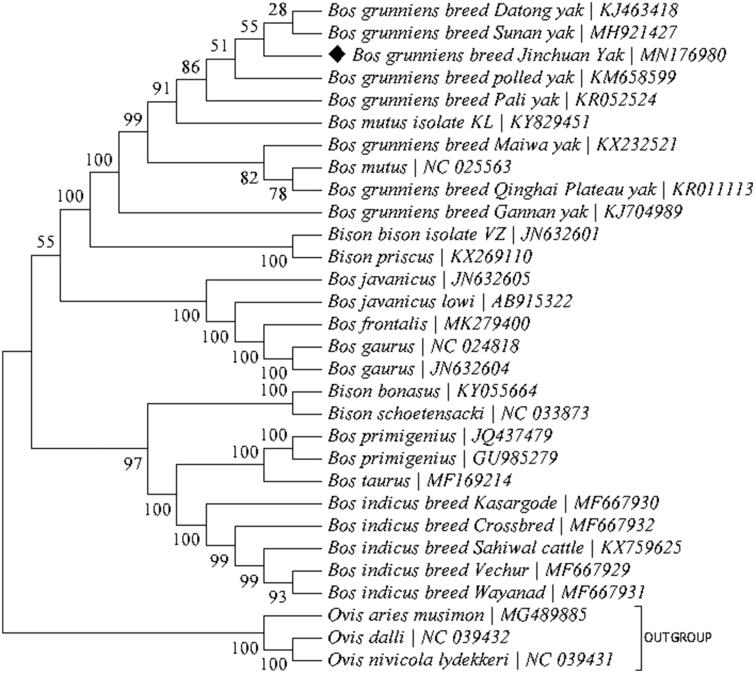
Phylogeny of two related genera Bison and Bos based on the maximum-likelihood analysis of the concatenated sequences of 13 mitochondrial protein-coding genes (alignment size: 11,370 bp). The best-fit nucleotide substitution model is ‘HKY＋G’. The support values next to the nodes are based on 400 boot-strap replicates.
